# Supplementary material for: Mapping the quality of Norwegian health information –Does it facilitate informed choices?
Source: PLoS One. 2026 Mar 2;21(3):e0327148. doi: 10.1371/journal.pone.0327148 (PMC12952600; doi:10.1371/journal.pone.0327148)
Supplement: S1 File — (DOC) [file pone.0327148.s001.doc]

STROBE Statement—Checklist of items that should be included in reports of ***cross-sectional studies***

|  | Item No | Recommendation |
| --- | --- | --- |
| **Title and abstract** | 1 | (*a*) Indicate the study’s design with a commonly used term in the title or the abstract  **P1 Line 1, P2 Line 39** |
|  |  | (*b*) Provide in the abstract an informative and balanced summary of what was done and what was found  **P2** |
| Introduction | | |
| Background/rationale | 2 | Explain the scientific background and rationale for the investigation being reported  **P3 and 4** |
| Objectives | 3 | State specific objectives, including any prespecified hypotheses  **P4 line 120-126** |
| Methods | | |
| Study design | 4 | Present key elements of study design early in the paper  **P4** |
| Setting | 5 | Describe the setting, locations, and relevant dates, including periods of recruitment, exposure, follow-up, and data collection  **P5 Lines 147-185 and P6 Line 2021 - P7 Line 252** |
| Participants | 6 | (*a*) Give the eligibility criteria, and the sources and methods of selection of participants  **P5 Lines 147-185** |
| Variables | 7 | Clearly define all outcomes, exposures, predictors, potential confounders, and effect modifiers. Give diagnostic criteria, if applicable  **P6 Lines 187-218** |
| Data sources/ measurement | 8* | For each variable of interest, give sources of data and details of methods of assessment (measurement). Describe comparability of assessment methods if there is more than one group  **P6 Lines 187-218** |
| Bias | 9 | Describe any efforts to address potential sources of bias  **P6 Line 221 – P7 Line 252** |
| Study size | 10 | Explain how the study size was arrived at  **P5 Line 173 - 176 there has been no power calculation** |
| Quantitative variables | 11 | Explain how quantitative variables were handled in the analyses. If applicable, describe which groupings were chosen and why  **Page 7 line 255 – Page 8 Line P6 285** |
| Statistical methods | 12 | (*a*) Describe all statistical methods, including those used to control for confounding  **Page 7 line 255 – Page 8 Line P6 285** |
|  |  | (*b*) Describe any methods used to examine subgroups and interactions  **Page 7 line 255 – Page 8 Line P6 285 ANOVAS for three factors** |
|  |  | (*c*) Explain how missing data were addressed  **Not relevant** |
|  |  | (*d*) If applicable, describe analytical methods taking account of sampling strategy  **Not applicable** |
|  |  | (*e*) Describe any sensitivity analyses  **Page 7 line 255 – Page 8 Line P6 285 ANOVAS for three factors** |
| Results | | |
| Participants | 13* | (a) Report numbers of individuals at each stage of study—eg numbers potentially eligible, examined for eligibility, confirmed eligible, included in the study, completing follow-up, and analysed  **P8 Lines 288 – 293** |
|  |  | **P8 Lines 288 – 293** |
|  |  | (c) Consider use of a flow diagram  **Not applicable** |
| Descriptive data | 14* | (a) Give characteristics of study participants (eg demographic, clinical, social) and information on exposures and potential confounders  **P8 Line 288 – 293 distribution over provider classes** |
|  |  | (b) Indicate number of participants with missing data for each variable of interest  **Tabel 1** |
| Outcome data | 15* | Report numbers of outcome events or summary measures  **P8 Line 300 – 305, Table 1, 2 and figure 1** the study is not differentiating exposed and unexposed groups |
| Main results | 16 | (*a*) Give unadjusted estimates and, if applicable, confounder-adjusted estimates and their precision (eg, 95% confidence interval). Make clear which confounders were adjusted for and why they were included  **Not applicable** |
|  |  | (*b*) Report category boundaries when continuous variables were categorized  **Table 1, 2 P8 Line 300 – 305** |
|  |  | (*c*) If relevant, consider translating estimates of relative risk into absolute risk for a meaningful time period  **Not relevant** |
| Other analyses | 17 | Report other analyses done—eg analyses of subgroups and interactions, and sensitivity analyses  **Page 12 Line 223 – 243 and table 3** |
| Discussion | | |
| Key results | 18 | Summarise key results with reference to study objectives  **P 13 Line 355-370** |
| Limitations | 19 | Discuss limitations of the study, taking into account sources of potential bias or imprecision. Discuss both direction and magnitude of any potential bias  **P 13 Line 371- P14 Line 411** |
| Interpretation | 20 | Give a cautious overall interpretation of results considering objectives, limitations, multiplicity of analyses, results from similar studies, and other relevant evidence  **P 14 Line 412 P 15 Line 497** |
| Generalisability | 21 | Discuss the generalisability (external validity) of the study results  **P13 Line 372-376** |
| Other information | | |
| Funding | 22 | Give the source of funding and the role of the funders for the present study and, if applicable, for the original study on which the present article is based  **P17 Line 519-521** |

*Give information separately for exposed and unexposed groups.

**Note:** An Explanation and Elaboration article discusses each checklist item and gives methodological background and published examples of transparent reporting. The STROBE checklist is best used in conjunction with this article (freely available on the Web sites of PLoS Medicine at http://www.plosmedicine.org/, Annals of Internal Medicine at http://www.annals.org/, and Epidemiology at http://www.epidem.com/). Information on the STROBE Initiative is available at www.strobe-statement.org.
