# Supplementary material for: Mapping the quality of Norwegian health information –Does it facilitate informed choices?
Source: PLoS One. 2026 Mar 2;21(3):e0327148. doi: 10.1371/journal.pone.0327148 (PMC12952600; doi:10.1371/journal.pone.0327148)
Supplement: Attachment — Submitted filename: point by point response letter.docx [file pone.0327148.s003.docx]

Submission ID [PONE-D-24-48366] - [EMID:b5b6dad108cf1c12]

Letter to the editor	November 30^th^, 2025

Dear Editors,

thank you for providing us the opportunity to improve our manuscript based on the reviewers’ feedback. The manuscript has now been language edited by Wiley Editing Services. We decided to attach the response letter from the language editors as a proof rather than uploading the manuscript with all track changes. Changes have been made across the whole document, and we do really appreciate this improvement step. However, the content of the manuscript has not been altered in any way.

We have made sure that our manuscript meets PLOS ONE's style requirements and made some small changes in the table formatting.

Thank you again.

Sincerely yours

Jürgen Kasper on behalf of the authors

**Reviewers' comments and our response:**

Comments to the Author

1. If the authors have adequately addressed your comments raised in a previous round of review and you feel that this manuscript is now acceptable for publication, you may indicate that here to bypass the “Comments to the Author” section, enter your conflict of interest statement in the “Confidential to Editor” section, and submit your "Accept" recommendation.

Reviewer #1: (No Response)

Reviewer #2: All comments have been addressed

2. Is the manuscript technically sound, and do the data support the conclusions?

The manuscript must describe a technically sound piece of scientific research with data that supports the conclusions. Experiments must have been conducted rigorously, with appropriate controls, replication, and sample sizes. The conclusions must be drawn appropriately based on the data presented.

Reviewer #1: Partly

Reviewer #2: Yes

3. Has the statistical analysis been performed appropriately and rigorously?

Reviewer #1: Yes

Reviewer #2: Yes

4. Have the authors made all data underlying the findings in their manuscript fully available?

The PLOS Data policy requires authors to make all data underlying the findings described in their manuscript fully available without restriction, with rare exception (please refer to the Data Availability Statement in the manuscript PDF file). The data should be provided as part of the manuscript or its supporting information, or deposited to a public repository. For example, in addition to summary statistics, the data points behind means, medians and variance measures should be available. If there are restrictions on publicly sharing data—e.g. participant privacy or use of data from a third party—those must be specified.

Reviewer #1: Yes

Reviewer #2: Yes

5. Is the manuscript presented in an intelligible fashion and written in standard English?

PLOS ONE does not copyedit accepted manuscripts, so the language in submitted articles must be clear, correct, and unambiguous. Any typographical or grammatical errors should be corrected at revision, so please note any specific errors here.

Reviewer #1: No

Reviewer #2: No

6. Review Comments to the Author

Please use the space provided to explain your answers to the questions above. You may also include additional comments for the author, including concerns about dual publication, research ethics, or publication ethics. (Please upload your review as an attachment if it exceeds 20,000 characters)

Reviewer #1: The manuscript is much improved with the concerns largely addressed. However, the manuscript is still not ready for publication. Language, while improved, still is not to academic standard. Passive voice is used throughout (e.g., "The quality of the measurement" (Line 341), "The quality of the information" (Line 349, etc. Some language is awkward (e.g., does it matter that Master's students were used? I don't think so, they're research assistants in my mind). Titles for figures also contain awkward phrasing. (e.g., "Fig 1: Title: Overview over the procedure of the multi-cross-sectional study"The tables are not publication quality and do not use standard formats for things like table footnotes (i.e., asterisk, daggers, etc.). While the STROBE statement is followed, the use of headings is not consistent. The paper refers to chapters, which is not correct.

On the whole, this is a much improved draft but it is still not ready for publication in an English-language journal. The major issues that remain are formatting and language, especially as PLOS ONE does not copy edit manuscripts.

Response: thank you for providing us this feedback. We used Wiley language editing service and received a thorough review of the language. We adopted all recommended changes. In addition, rephrased the titles of the figures and legends, removed a couple of subheadings and also slightly changed the tables’ format.

This said, the work is interesting and the findings are reasonable given the methods. Certainly, to find out that most health information doesn't meet minimum standards is a worthwhile finding deserving of publication. A further reworking of the text should get the job done.

Reviewer #2: Unfortunately, I was unable to find the letter with point-by-point comments to the questions raised. However, while reviewing the edited manuscript I see much has been changed by the authors and especially the methodology is more clear. The manuscript is dense and lengthy, some of the additional text provided helps to offer clarity in some sections, but the text could benefit from copy editing. Some of the sentences have grammatical errors or are difficult to read.

Response: thank you, we have addressed these problems in our new revision.

7. PLOS authors have the option to publish the peer review history of their article (what does this mean?). If published, this will include your full peer review and any attached files.

If you choose “no”, your identity will remain anonymous but your review may still be made public.

Do you want your identity to be public for this peer review? For information about this choice, including consent withdrawal, please see our Privacy Policy.

Reviewer #1: No

Reviewer #2: Yes: Andrea Solnes Miltenburg
