# Supplementary material for: Mapping the quality of Norwegian health information –Does it facilitate informed choices?
Source: PLoS One. 2026 Mar 2;21(3):e0327148. doi: 10.1371/journal.pone.0327148 (PMC12952600; doi:10.1371/journal.pone.0327148)
Supplement: Attachment — Submitted filename: point by point response R3.docx [file pone.0327148.s004.docx]

Submission ID [PONE-D-24-48366R3] - [EMID:15acfda3b72fea1f]

Letter to the editor	January 8^th^, 2026

Dear Editors,

thank you for providing us the opportunity to improve our manuscript based on the reviewers’ final suggestions, which were quite useful. Apart from one suggestion, we have implemented all of them.

Thank you again.

Sincerely yours

Jürgen Kasper on behalf of the authors

**Reviewers' comments and our response:**

Comments to the Author

Reviewer #1: 6. Review Comments to the Author

Reviewer #1: Thank you for the opportunity to review this final manuscript revision. Congratulations on the effort required to revise this work. I appreciate the efforts of the authors to incorporate the suggestions of the peer reviewers.

Response: Thank you for this feedback.

My concerns about the last version were rooted in readability – these have largely been addressed. I note a few minor issues that I’ll mention here to help the authors finalize their manuscript before final consideration is given by the PLOS editorial group.

Response: We are grateful to the reviewer for once again thoroughly reading through our manuscript.

Line 61…delete “and permitted” as I think this word choice introduces a bunch of ideas that I don’t think are needed here (i.e., patient autonomy) that I think would warrant more discussion to fully address. Suggest leaving it out.

Response: Thank you, we have removed “and permitted”.

Line 64…replace “enable” with “enabling” for correct verb tense.

Response: Thank you, we changed “enable” to “enabling”.

Line 68…delete “the” in front of “informed choice”, it’s not necessary.

Response: Thank you, we removed “the”.

Line 75…suggest deleting ‘, or even most,’ as it is distracting and doesn’t add anything.

Response: thank you, we removed the phrase.

Line 108…delete ‘even’, it’s a bit conversational and not needed.

Response: We removed the word “even”.

Line 111…consider capitalizing Public Health Nurses before the acronym and drop the ‘s’ from (PHNs) as it’s not needed in the bracketed acronym.

Response: Thank you. We changed the term according to you suggestion.

Line 122…might want to soften this sentence to say “To the best of our knowledge, this type of research has not been conducted in Norway….”

Response: No problem, we changed this sentence

Line 146…the sentence beginning with “Focussing on the reality….” needs revision as it reads awkwardly. Suggest “We designed this study from the user’s perspective given that it is the individual who seeks out health information in the majority of cases and thus to whom we want to generalize our results.” or something to that effect. It’s the right idea, just needs a bit of revising.

Response: thank you, we inserted your suggested sentence.

Line 205 – 209…the authors may wish to consider alternative formatting…numbering the items in text might be better than calling them out as a numbered list.

Response: thank you, we used “first,”, “second,” “third,” now.

Line 251…delete (MS) as it’s not relevant, Excel is not all capitalized and include the version number that was used. Software citations do follow a format, usually along the lines of ‘Ratings were documented in Excel Version 2504 (Microsoft Corporation)…”

Response: ok, thank you. We corrected the writing and deleted the “MS”. As we even provide a publication reference to the tool, we did not further detail the version.

Line 318…this probably belongs in a footnote or elsewhere in text; it’s kind of hanging out on its own and it’s not clear what the data file is.

Response: Thank you for this good idea which we implemented

Line 344…the research questions should be introduced in the Introduction. They read awkwardly here. Revision is necessary.

Response: Thank you for this comment. The intention was to provide a reminder of the three research questions to the reader. We shortened and rewrote these introductions to the particular result sections to avoid them looking like the first mention of the research questions. In addition, we numbered the research questions in the paragraph on the aim of the study.

Line 370….strip out the bracketed text in the heading, the title label should speak for itself.

Response: ok

Line 425…extra space between ‘the’ and ‘target’ needs to be deleted.

Response: done

Line 428…reference to Google should probably be broader to something that includes search engines and indeed, large language model AI tools like ChatGPT.

Response: thank you, we added this broadened view.

Line 469…this section needs to be before limitations.

Response: Thank you for this suggestion. Well, there are different ways to structure the discussion. We choose to present and discuss limitations before the broader context. This decision is based on a psychologic consideration according to which the reader is more open for the discussion of the meaning of the results after potential concerns or shortcomings are worked off. We want to keep this structure.

Line 555…Suggest replacing “Regardless” with “Independent” as it has a bit more of a scientific flavour to it.

Response: done

Overall, this is a much-improved manuscript. I think these suggestions are largely cosmetic at this point. Congratulations again to the authors on piece of work that answers a really relevant question about health information available online.

Response: Thank you very much for your work and support.
